# Participative development and evaluation of a communication skills–training program for oncologists—patient perspectives on training content and teaching methods

**DOI:** 10.1007/s00520-021-06610-1

**Published:** 2021-10-09

**Authors:** Nicole Ernstmann, Hannah Nakata, Lena Meurer, Johanna Weiß, Franziska Geiser, Frank Vitinius, Andrea Petermann-Meyer, Markus Burgmer, Bernd Sonntag, Martin Teufel, André Karger

**Affiliations:** 1 Department for Psychosomatic Medicine and Psychotherapy, Center for Health Communication and Health Services Research (CHSR), University Hospital Bonn, Bonn, Germany; 2 Center for Integrated Oncology Aachen, Bonn, Cologne, Düsseldorf (CIO ABCD), Germany; 3 Department for Psychosomatic Medicine and Psychotherapy, University Hospital Bonn, Bonn, Germany; 4 Department of Psychosomatics and Psychotherapy, University of Cologne, Faculty of Medicine and University Hospital Cologne, Cologne, Germany; 5 Department of Oncology, Hematology, Hemostaseology and Stem Cell Transplantation, Medical Faculty, University Hospital RWTH Aachen, Aachen, Germany; 6 Department of Psychosomatics and Psychotherapy, LWL-Hospital Münster and University Hospital of Münster, Münster, Germany; 7 Department of Psychosomatic Medicine and Psychotherapy, LVR University Hospital Essen, University of Duisburg-Essen, Essen, Germany; 8 West German Cancer Center (WTZ), University Hospital Essen, Essen, Germany; 9 Clinical Institute of Psychosomatic Medicine and Psychotherapy, Medical Faculty, University Düsseldorf, Düsseldorf, Germany

**Keywords:** Communication skills training, Oncology, Cancer patients, Formative evaluation

## Abstract

**Background:**

Using the 6-step approach to curriculum development for medical education, we developed a communication skills training (CST) curriculum for oncology and evaluated this curriculum from the perspective of cancer patients.

**Methods:**

We conducted a qualitative interview study with cancer patients, collecting data using semi-structured face-to-face or telephone interviews with a short standardized survey. We fully transcribed the audiotaped interviews and conducted the content analysis using MAXQDA 2020. We analyzed the quantitative sociodemographic data descriptively.

**Results:**

A total of 22 cancer patients participated, having a mean age of 60.6 (*SD*, 13.2) years and being predominantly female (55%). The patients believed that the CST curriculum addressed important aspects of patient-centered communication in cancer care. They emphasized the importance of physicians acquiring communication skills to establish a trusting relationship between doctor and patient, show empathy, inform patients, and involve them in treatment decisions. The patients had some doubts concerning the usefulness of strict protocols or checklists (e.g., they feared that protocol adherence might disturb the conversation flow).

**Discussion:**

Although it was a challenge for some participants to take the perspective of a trainer and comment on the CST content and teaching methods, the patients provided a valuable perspective that can help overcome blind spots in CST concepts.

## Background

The goal of patient-centered communication is to provide care that is concordant with patients’ values, needs, and preferences and allow them to participate actively in decisions concerning their health and health care [1]. In oncology, patient-centered communication should support cancer patients in establishing a trusting relationship with their physicians, handling the emotional impact of a life-threatening illness, managing complex information, making treatment decisions, and dealing with uncertainty while maintaining hope [2–5].

One way for physicians to support patient-centered communication is by using communication skills training (CST) programs. Guidelines and recommendations exist for core components and didactics that emphasize participant-centered skills training in small groups with multisource feedback and evaluation [4–6]. Reviews have shown that CST in oncology can be useful and effective for physicians’ direct evaluation, skills, behaviors, attitudes, or self-efficacy (e.g., [7–11]). Effectiveness remains inconsistent for the effects of CST over time and for intermediate and long-term patient-reported outcomes [10–12].

Cancer patients’ experiences and their evaluation of curriculum concepts might be of great value in the CST development process to better achieve the mission of patient-centered communication. Some studies have integrated survey results for cancer patients’ communication preferences into curriculum development [13]. Sinclair et al. have examined the perspectives of patients with advanced cancer on the health care providers’ compassion training [14]. However, in developing CST for oncologists, the patient perspective has not been incorporated thus far [15].

## Aim

This study evaluates a CST curriculum for oncologists from the cancer patients’ perspectives. The main objective of this study is to gather patients’ views on training content and teaching methods.

## Methods

### Development of a CST for oncologists

Using the 6-step approach for curriculum development for medical education [16], we developed a CST [17] for physicians at 5 comprehensive cancer centers in North Rhine, Westphalia, Germany. As part of the needs assessment, we conducted a survey to incorporate oncologists’ preferences into the development [18]. The CST comprises 4 modules; its topics and teaching methods are shown in Table 1.

**Table 1:** CST curriculum topics and teaching methods

Module	Unit	Topic/section	Teaching methods
A. Delivering Bad News	1	Challenges to successful communication	Back-to-back drawing (the instructor has to get the partner to draw a duplicate of a picture using only verbal directions). Group discussion
		Appropriate and inappropriate expressions when delivering bad news	Group discussion about a video on breaking bad news
		Challenges in delivering bad news	Exchange of experiences and a collection of “dos and don’ts.”
	2	Active listening	Small-group role-play and observation
		Delivering bad news	Use of a structured reporting form to collect case studies experienced by the participants as a basis for later role-plays
		SPIKES–6-step protocol for delivering bad news [19]	Lecture
			Role-play with trainer and actor patient
	3	SPIKES–6-step protocol for delivering bad news [19]	Role-play with participants and actor patients. Video and small-group feedback
			Role-play based on case studies reported by the participants, with participants playing the doctor and patient roles. Feedback provided by participants
	4	Reflection	Reflection in small groups, with a focus on helpful techniques and transfer into practice
			“Pack your bag”– Collection of individual take-home messages
B. Shared Decision-Making	1	Reflection on Module A	Reflection in small groups, focusing on helpful techniques in practice
		Difficulties in decision-making	Use of a structured reporting form to collect case studies experienced by the participants as a basis for later role-plays. Reflect on factors impeding the decision-making process
		Shared decision-making: team, option, and decision talks [20, 21]	Lecture
	2	Shared decision-making: option and decision talks	Role-play among participants to practice option talk and team talk using a car repair example
		Shared decision-making: Team, option, and decision talks	Role-play among participants based on the participant case studies. Feedback provided by participants. Group discussion
	3	Shared decision-making: Team, option, and decision talks	Role-play with actor patients. Feedback in small groups
		OPTION scale [22, 23]	Introduction of a scale measuring the involvement of patients in decision-making for the observation of role plays
	4	Shared decision-making	Role-play with actor patients
		Reflection	“Pack your bag”–Collection of individual take-home messages
C. Talking about Death and Dying	1	Reflections on Modules A and B	Reflect on helpful techniques in practice
	2	NURSE model [24, 25]	Discussion and development of the elements of the NURSE model
	3		Best-practice video and discussion
	4		Discussion about emotionally challenging situations in a role-play with participants
	5		Role-play with actor patients at the beginning of their palliative care. Feedback provided by actor patients and participants
D. Team Communication	1	Reflection on Modules A, B, and C	Reflect on helpful techniques in practice
		Dealing with relatives	Discussion in groups of 2 participants on challenges in dealing with relatives
		Communicating with relatives	Lecture and fact sheet
		Dealing with emotions BUSTER Protocol [26]	Lecture
		Dealing with relatives	Development of standards for dealing with relatives in small groups
		Family talk	Development of a standard operating procedure based on a case study
			Role-play with participants playing the doctor, patient, partner, and children roles
	2	Team communication	Collection of challenging team communication case studies experienced by the participants as a basis for later role-plays
			Sociodramatic role-play to involve all participants
		Reflection	Individual take-home messages
	3	Taboos of making mistakes	Lecture with the video “Physicians Make Mistakes.” Group discussion on the safety culture in medicine
		Errors	Exchange of experiences. Reflection on one’s attitude toward mistakes
		Open disclosure, second victim, patients’ needs, ethics of open disclosure	Lecture
	4	Open disclosure	Role-play on communicating with patients and relatives
		Legal basis of error communication	Lecture
		Reflection	Reflect in small groups, with a focus on helpful techniques in practice

### Study design, sampling, and recruitment

We conducted our qualitative interview study in 2018–2020. The Ethics Committee of the Medical Faculty of the University of Bonn approved the study. The convenience sampling criteria included patient accessibility and willingness to participate. The purposeful sampling criteria included patient age and gender. The goal was to include persons of both sexes differing in age.

We recruited the participants from clinics from a comprehensive cancer center, via cancer support groups, and by telephone recruitment of participants in previous surveys. Student assistants conducted the screening interviews in the waiting area of the cancer center. They consecutively asked any person present. The inclusion criteria for survey participation were a confirmed cancer diagnosis and written informed consent. The exclusion criteria were aspects that made it difficult to set up the interview (e.g., speech or comprehension problems, psychosis, dementia, pain, difficulties in concentrating) or withdrawal of consent. After receiving the written informed consent, the interviewers contacted participants by phone to arrange the interview date and mode.

### Data collection

We collected data using semi-structured face-to-face or telephone interviews. Three female psychologists and one female social worker conducted and audiotaped the interviews. The face-to-face interviews took place at the cancer center, in the researchers’ office, or at the participants’ homes. The interviewers took field notes during and after the interviews. The participants completed a short standardized questionnaire with factual sociodemographic and illness-related items (i.e., age, sex, marital status, education, cancer diagnosis, time since diagnosis, cancer treatment) before the interview.

The interdisciplinary research team developed the interview guidelines for Modules A, B, and D. Module C was not part of the interview study. The interviewers felt that the emotional burden on the study participants would be too heavy when confronted with death and dying since some of the participants were interviewed during their acute cancer treatment. We pretested the interview guidelines for Module A in 2 cognitive interviews, focusing on comprehensibility and acceptability, subsequently revising them.

The guidelines began with initial narrative questions addressing the patient’s own experiences and communication needs in medical encounters (e.g., “What did you experience in conversations between your doctor and your relatives?”). Open questions addressed relevant program topics for a specific CST module (e.g., “What, in your opinion, should be conveyed to the participants to enable them to deal with errors?”). For the patients’ perspectives on training content and teaching methods, we explained each topic and method first, then assessed the patients’ views of a topic’s relevance and the teaching method’s appropriateness. We supplemented the descriptions of the teaching methods with illustrative photos of each. Finally, we asked the patients to explain their answers.

The closing questions addressed missing aspects of the CST curriculum in terms of relevant topics or suitable teaching methods (e.g., “In your opinion, were important aspects missing that the doctors should have conveyed?”). The interviews lasted 40–126 min, with a mean duration of 64 min. One patient terminated the interview early because of attention problems.

### Data analysis

A transcription bureau fully transcribed the audiotaped interviews using transcription standards for social research [27]. The research team anonymized the transcripts. We conducted the content analysis using MAXQDA 2020 and read the transcripts multiple times. The two researchers who conducted the interviews coded the data deductively. They discussed ambiguous and diversely determined codes and added more inductive categories. We coded 1068 text units, reducing the data and linking the codes to direct quotes. The research team discussed the codes and final categories. We analyzed the quantitative data descriptively using IBM SPSS Statistics version 26.

## Results

### Sample characteristics

Of the 55 screened cancer patients, 13 patients refused to participate, and 22 cancer patients agreed to participate (response rate 63%). The mean patient age was 60.6 (*SD*, 13.2) years, and 55% were female. The most prevalent diagnosis was breast cancer, and the mean time since diagnosis was 7.1 (*SD*, 6.4) years. Most participants received chemotherapy and surgery. Table 2 provides a detailed description of the patient characteristics.

**Table 2:** Patient characteristics

Characteristic	*N*	%	Mean *(SD)*
Age			60.6 (13.2)
Time since diagnosis (years)			7.1 (6.4)
Sex	22	100	
Male	10	45.5	
Female	12	54.5	
Marital status	22	100	
Married/in partnership	17	77.3	
Divorced	3	13.6	
Widowed	0	0	
Single	2	9.1	
Education	22	100	
No lower secondary school education	0	0	
Lower secondary school education	1	4.5	
Intermediate secondary school education	2	9.1	
Subject-related entrance qualification	3	13.6	
General qualification for university entrance	1	4.5	
Vocational education	4	18.2	
University of applied science degree	5	22.7	
University degree	5	22.7	
Doctor’s degree	1	4.5	
Cancer diagnosis	22	100	
Breast cancer	9	40.9	
Lymphoma	5	22.7	
Prostate cancer	3	13.6	
Other	5	22.7	
Cancer treatment	73	100	
Chemotherapy	22	30.1	
Surgery	18	24.7	
Radiation	17	23.3	
Hormonal therapy	6	8.2	
Antibody therapy, immunotherapy	8	11.0	
Stem cell therapy	2	2.7	

### Patients’ perspectives on training contents

In the following sections, we summarized the results for each topic separately, using selected anchor examples. Study identification numbers appear in parentheses following each example.

#### Negative experiences and the role of CST for patient-centered care

All participants expressed the opinion that CST is useful and, in particular, is an important intervention to increase patient-centered care. The patients generally regarded empathetic communication skills and the establishment of a trusting patient–provider relationship as important. However, some patients expressed doubts as to whether such a complex CST is feasible in routine care.

Many patients described the negative experiences haunting them for a while as a reason for their belief that empathetic conversation is important. These experiences include bad news delivered casually and without showing compassion, ignoring their questions or information needs, talking about them in their presence, disregarding their concerns or fears, not explaining findings or therapies, not listening to them, and lacking authenticity.

“And they were always talking like that. And then I would ask, ‘Stop, what was that?’ You heard something, but they didn’t respond; they just kept going.” (1-2-LM)

#### Delivering bad news

The patients rated learning and practicing delivering bad news using the SPIKES protocol as an important CST element. The patients felt that the SPIKES protocol could help prepare for the conversation, build a trusting relationship between doctor and patient, avoid misunderstandings, and manage emotions. Trust is an important prerequisite for cancer treatment. The patients also rated pausing and active listening as important, with many regarding active listening as a central prerequisite for a successful conversation.

B: “[...] active listening is the direct contact, I think, between the doctor and me. And I think that’s extremely important in this situation. He has to signal to me, ‘I am now with you and your problems and nowhere else.’

I: Why do you think that it is important?

B: Because it’s a very sensitive situation where someone who does not feel that he is in good hands, who does not feel well addressed, can easily get into a crisis and can quickly lose heart and the confidence to make the best out of it.” (1-3-LM)

“‘Wait for the patient’s reaction.’ That’s a good idea. That I can first take a deep breath when getting such a diagnosis, or even cry, right?” (1-4-LM)

However, some patients believed that SPIKES was not feasible. They worried that the protocol steps were difficult to follow and that following a strict protocol would increase alienation between doctor and patient. Moreover, some patients felt that the invitation step could cause some confusion.

“‘How […] would you like to be informed […]?’ […] I think this would affect me, I get scared, he just says as much as I ask […]. And not what is really important, because I can’t judge that anyway.” (1-4-LM)

#### Shared decision-making

Most patients generally agreed with the mission and guiding principles of shared decision-making. For some participants, shared decision-making has the potential to enable them to cope with their illness better. Most rated the technique of reflecting on difficult decisions as positive and emphasized the importance of changing perspectives (i.e., asking what is difficult from the patient’s point of view). Reflection could be helpful to illustrate patients’ overload or helplessness when confronted with a difficult or far-reaching decision. The patients appreciated that the discussions might be difficult emotionally and factually. However, some participants questioned the importance of a reflection on difficult decisions.

“I don’t think that it is so important, to deal with it like that, to ask if it is a difficult decision or not. So the situation or the decision dictates it, […] whether this is a difficult decision, doesn’t it?” (2-1-LM)

The patients rated the 3-talk model of shared decision-making (i.e., team talk, option talk, and decision talk) [23, 24] as important for providing structure, exploring patients’ preferences, and reducing the probability of forgetting central aspects (e.g., discussing alternatives during the option talk), as well as potentially disturbing the conversation flow.

“I think that’s very important because the probability of making errors is probably minimized. If I don’t have a plan, or just sort of, I may forget some points, and that may then have a negative impact on the patient’s decision making.” (2-2-LM)

Regarding team talk to elicit patient goals, some patients worried about their active role, believing that they could be overwhelmed. In contrast, other patients expressed that team talk is an important aspect of a patient-centered, shared decision-making process. The patients also evaluated option talk positively, emphasizing that both benefits and disadvantages of the options are explained. Another positive aspect was differentiating between reliable information and unreliable or biased information from other media. The main advantage of decision talk is that patients are allowed time to think about their decision.

“I know there is no pressure that I have to do it today, that I have to make the decision today. […] And that gives you, um, a feeling of ‘it’s not essential now.’ Because cancer is always about death and dying. And thinking about it makes you feel like you have the time.” (2-1-LM)

#### Communication with relatives and team members

The participants viewed the inclusion of relatives as positive, as an opportunity to empower relatives to deal with the disease. Using relatives as a source of patient social support is strengthened by this empowerment, both emotionally and instrumentally. The patients viewed relatives as “fellow fighters” (4–8-JW). They also saw their own family members’ burdens and the need for physicians to acknowledge and respond to their support needs as well.

“But I think that’s also very, very important, because I notice that he’s also alone in this. […] he wants to play it cool with me: ‘So we can do this, right?’ But I notice that he is looking for a timeout […]. But a doctor hasn’t talked to him about it yet.” (4-4-LM)

The participants viewed the BUSTER protocol (Be prepared, Use non-judgmental listening, Six-second rule, “Tell me more” statements, Empathize and validate, Respond with a wish statement) [28] as a chance for physicians to build or rebuild relationships with patients and their families. Some patients believed that the protocol would be easy to adhere to and could help physicians prepare for challenging conversations and give patients the feeling of being respected and accepted. However, other patients doubted whether empathizing is always helpful for patients or relatives, believing that empathy or compassion could increase patient suffering.

“I would describe it positively, well, I wouldn’t say: ‘It’s not easy.’ [...] ‘Oh, you poor people, and how do you deal with it?’ I wouldn’t do that.” (4-4-LM)

The participants expressed only positive aspects for learning to communicate in an interprofessional team. The patients had requested interprofessional care, viewing it as a prerequisite for safe, high-quality health care. The patients also believed that improving team communication can strengthen mutual trust within the team.

“I can’t imagine being a patient and hearing something from a nurse that hasn’t been discussed with the doctor, or vice versa. It has to match.” (4-1-LM)

#### Error communication

The participants supported the idea of a group discussion on the taboo of making mistakes, believing that physicians are human beings and are prone to errors due to work pressure or a lack of experience. The patients regarded learning to talk about errors or adverse events as important, viewing errors as an opportunity to learn. The patients believed that disclosing errors to them would be a confidence-building measure that could have an empowering effect because physicians and patients were communicating at the same level.

“I think that someone who […] thinks he doesn’t make any mistakes, I wouldn’t want someone like that to treat me. Because if someone makes a mistake and thinks, ‘I’m faultless,’ then he tries to cover it up and makes everything much worse.” (4-4-LM).

The participants rated the lecture on the ethics of open disclosure as important. The patients thought that physicians should know about their rights and obligations. Some patients believed that today physicians often are obliged to keep errors or adverse events confidential. The preference or the readiness to admit an error or for open disclosure is a personal trait rather than part of an organization’s error culture. In cases of open disclosure, the patients perceived that physicians need the communication skills to be prepared for these conversations.

“That is very important for the doctors and so on, the nursing staff. They have to know how to formulate something, don’t they? You can either trigger aggression or acceptance through the wording alone, right?” (4-8-JW)

The participants regarded most steps in the open-disclosure process as helpful for guiding conversations. In particular, they considered this confidence-building if external contact persons were named for further questions or concerns. However, some patients queried the appropriateness of the question, “What can we do for you?”.

“If he offers me, ‘What can I do for you?’ He’ll cut off my right leg instead of my left, and then comes after I wake up from the anesthesia: ‘That went bad, I’m sorry. Is there anything else I can do for you?’ ‘Yes. Please don’t cut off the left one, too, right?’ [...] That’s a bit stupid.” (4-6-SJ)

The patients believed that, once an error occurs, health professionals should examine the error chain carefully to learn from and prevent errors in the future. Most patients emphasized the organizational and systemic factors leading to errors. The patients rated the topic of the second victim as important and helpful for health professionals.

“If [errors] happen often, then something is wrong. Not because the person is somehow stupid or something, but because there is something wrong with the system. Then […] a senior physician [should be there] to control it so that structures can be changed so that this can no longer happen. Because nobody does it on purpose.” (4-2-LM)

“[...] staff, doctors, nurses [...] should not take the patients’ diagnoses home, ideally, and should also leave their own actions in the clinic and not take them home. Because that’s even more of a burden when I take it home. That promotes burnout, stress, depression, all of which, if I can’t switch off and don’t have a contact person. That is why it is important that this is discussed.” (4-6-SJ)

### Patients’ perspectives on teaching methods

The participants’ perspectives reveal the benefits associated with each teaching method. The patients considered it valuable if examples and daily clinical experiences were included. In general, the patients considered practicing communication skills in role-playing suitable; however, some associated negative aspects with certain methods. Some missed aspects of non-verbal communication, especially techniques to pay attention to non-verbal signals from patients. Furthermore, the patients questioned the role-play to practice team talk using the example of a car repair as not being transferable to medical care. In addition, some patients doubted whether physicians would dare to speak about their own errors in a group.

“I find that difficult now. Because there has to be a lot of trust among this group of doctors in order to be really honest. Who likes to admit their mistakes in front of colleagues and then be told what they did wrong?” (4-2-LM)

The participants suggested improvements to or further development of the methods. Some recommended using real patients instead of actor patients, believing that actors cannot adequately portray a cancer patient receiving bad news. The patients also considered constant repetition or refresher courses important to consolidate their communication skills.

“What I’ve just seen is more of a start, right? You discuss it once. But you would just have to repeat it more often or with another disease. [...] It doesn’t have to be a difficult case, right? [...] So that this, that they become familiar with it, with this conversation technique, that would be good.” (2-4-LM)

Some patients reflected on the interdisciplinary aspect of cancer care and emphasized the importance of teaching interprofessional teams.

“I think it’s so, so important, that all professions, at least the people closest to the patient, are included […].” (4-2-LM)

## Discussion

The aim of this study was to evaluate the curriculum of a CST for oncologists from the cancer patients’ perspective. Our results show that the participants believed that the CST curriculum addresses important aspects of patient-centered communication in cancer care. Patients valued the idea of teaching physicians how to communicate and of supporting them in improving their communication skills, especially in managing challenging situations. They underlined the importance of physicians acquiring communication skills to establish a trusting relationship between doctor and patient and to show empathy to meet patient communication needs and preferences. Previous research on whether the SPIKES protocol was consistent with patient preferences had similar results, demonstrating that the existing guidelines for breaking bad news largely reflected cancer patients’ perspectives [29].

Participants also supported teaching physicians how to inform cancer patients and involve them in decision-making. However, some patients were concerned that taking an active role might overwhelm them. This finding matches that from prior research on cancer patients’ communication preferences, suggesting that shared decision-making as a practice standard must be balanced against individual preferences [30]. Other patients expressed concerns about the side effects of strict protocols, fearing that the guidelines would either be difficult to follow or result in an increasing distance between doctor and patient or more difficulty reacting to patients’ individual needs and concerns.

This analysis revealed that the patients supported the idea of learning to meet the communication needs of caregivers and their families. The patients had expected interprofessional health care and, therefore, emphasized the importance of team and error communication. Open disclosure was consistently regarded as an important element of a trusting patient–physician relationship.

The current results show a mixed picture of patient perspectives on teaching methods. The patients viewed benefits in every method, as well as saw limitations or barriers to success. In general, methods incorporating interactive elements and real patient experiences were most valuable. Using actor patients was problematic, with many patients believing that actors cannot portray emotions as real patients can.

### Limitations

Several methodological issues must be considered when interpreting these results. Our qualitative research findings do not represent all cancer patients; generalizability is limited to the subjective appraisal of the participating patients. As in many health services research studies, our study design led to a selection bias, in this case for native-German-speaking, higher educated, and motivated patients. The recruiting methods resulted in a final patient sample that was either undergoing acute therapy or had been diagnosed several years ago. Patients in advanced stages are underrepresented in the sample, although a few patients had experienced recurrence or metastasis. Patients of advanced age, in advanced cancer stages, with cognitive impairments, or having poor language skills might experience more challenging medical encounters and therefore might have provided divergent results. Future studies also should address vulnerable patient groups.

Our interview guideline was semi-structured, with questions closely related to the developed curriculum. We combined an open narrative beginning with a more structured interview using the CST elements. Narrative elements based on the patients’ own preferences and experiences might have been hindered in the latter parts of the interviews. The participants easily changed their perspective and showed empathy for the physicians’ situation and their professional challenges, including work pressure or lack of experience or time. However, some participants found it challenging to take the trainer’s perspective and comment on the teaching methods. In addition, some patients may have had difficulties imagining a role-play with actor patients.

### Implications

The participative approach is the main strength of our work. By incorporating the patients’ perspectives, we aimed to overcome blind spots (e.g., missing contents or missing medical encounters in cancer care as perceived by patients) regarding routinely referring to well-known topics, and we evaluated didactic approaches to CST. Our results underline the importance of patient-centered communication and trusting relationships in cancer care. Hence, CST should incorporate the essential elements of clinical communication: introspection, reflexivity, and relational aspects of the clinical encounter [18].

Moreover, patients’ perspectives have proved to be valuable and illuminating in the CST development process. It was encouraging to integrate patients’ and survivors’ perspectives and experiences in the CST curricula as role players, patient advocates, or feedback providers in future interventions. In the next step, we will discuss these role extensions in the research team and integrate suitable aspects into future revisions of the CST. In future research, these revised CST will have to be subject to comprehensive process evaluation and feasibility testing as we cannot predict the burden associated with these task for patients and survivors.

New CST concepts are continuously being developed. One emerging trend is to include patients’ experiences [28, 31]. Based on our own experience, we recommend including the patient perspective in the CST content development and the development of innovative CST concepts.

## Data Availability

According to the patient consent form, the interview data is not available for scientific use by anyone other than the project’s group members.
